# Myocardial contrast echocardiography in the diagnosis of postoperative takotsubo myocardiopathy: case report and literature review

**DOI:** 10.1186/s12872-018-0985-z

**Published:** 2019-01-08

**Authors:** Jia-Hui Zeng, Wei Li, Feng-Juan Yao, Dong-Hong Liu, Cui-Ling Li, Yan-Qiu Liu, Rui Fan, Min Ye, Hong Lin

**Affiliations:** Department of Medical Ultrasonics, The First Affiliated Hospital of Sun Yat-Sen University, No. 58, Zhongshan Road 2, Guangzhou, 510080 People’s Republic of China

**Keywords:** Takotsubo cardiomyopathy, Myocardial contrast echocardiography

## Abstract

**Background:**

Takotsubo cardiomyopathy (TCM) is a brief ventricular dysfunction that usually occurs after emotional or physical stress. Here, we report a patient who underwent cardiac surgery and then developed TCM during the postoperative period.

**Case presentation:**

A 51-year-old woman was admitted to our hospital complaining of chest tightness, palpitations and dyspnoea after activity. An echocardiogram performed by our hospital showed rheumatic heart disease (severe mitral stenosis and regurgitation) with normal cardiac function and wall motion. After mitral valve replacement, this patient developed heart failure with low blood pressure and tachycardia. Urgent bedside echocardiography demonstrated akinesis in the middle and apical segments of the left ventricle and a depressed ejection fraction (EF) of 36%. Myocardial contrast echocardiography (MCE) showed similar enhancement intensity in the basal, middle and apical segments. Quantitative analysis showed approximately equivalent maximum intensity in these regions. The diagnosis was considered TCM instead of myocardial infarction. Then, an intra-aortic balloon pump was inserted to maintain effective circulation and reduce the postcardiac load. Given ventilation therapy, postoperative anticoagulation therapy and anti-infection treatment, the patient recovered quickly. In the follow-up examination, the patient remained asymptomatic and showed normalization of ventricular wall motion in the apical segment.

**Conclusion:**

This report presents a case of TCM in which MCE was used to demonstrate intact microvascular perfusion despite apical akinesis. This report might support the use of MCE as a substitute for invasive coronary angiography.

## Background

Takotsubo cardiomyopathy (TCM) is a brief ventricular dysfunction that occurs in the absence of obstructive coronary artery disease (CAD) [1]. TCM derives its name from the end-systolic shape of the left ventricle seen on left ventriculogram, which is similar to that of a Japanese octopus trap, and typically involves apical akinesis and compensatory basal hyperkinesis. Except for the typical form, a few cases of atypical (i.e. inverted) TCM have been described as well [2].

**Fig. 1:**
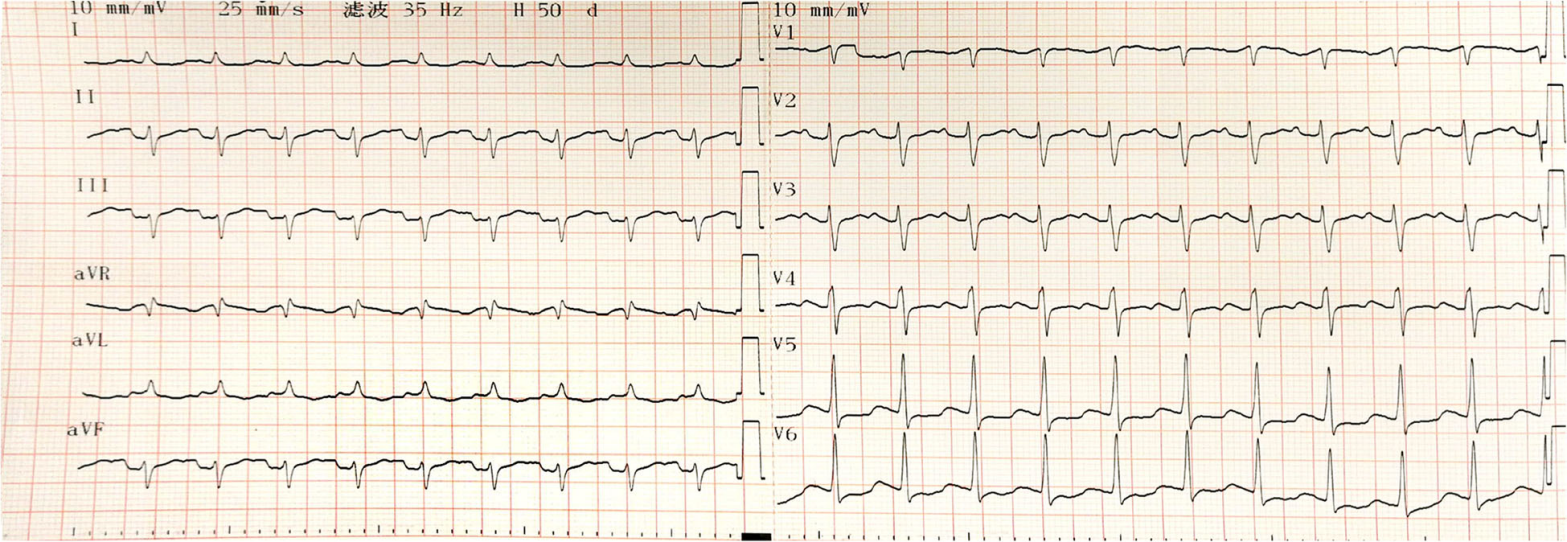
Electrocardiogram at the time this patient had sudden heart failure. V5–6 ST-segment depression was 0.1 mV

**Fig. 2:**
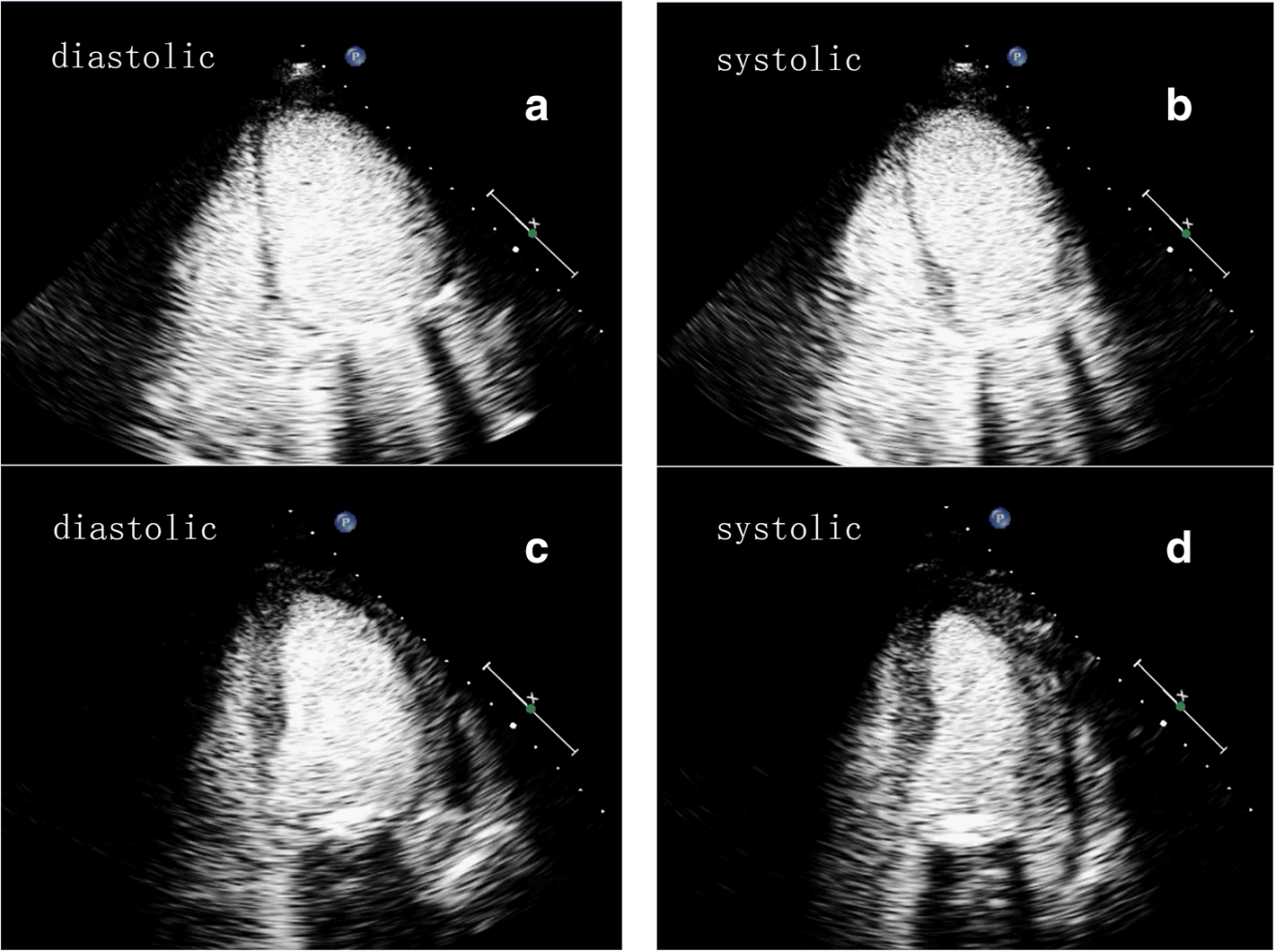
Left ventricular opacification (LVO) performed on the second day after surgery (**a** and **b**) and two weeks later (**c** and **d**). LVO performed on the second day after surgery (**a** and **b**) showed akinesis in the apical and middle segments of the left ventricle in the four-chamber view. LVO performed two weeks later (**c** and **d**) showed that apical movement was significantly improved with a slight decrease in interventricular septal motion

**Fig. 3:**
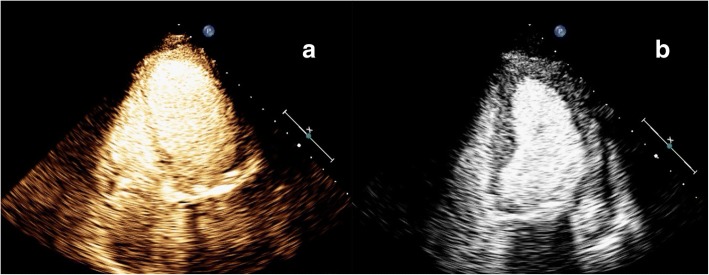
Myocardial contrast echocardiography (MCE) performed on the second day after surgery (**a**) and two weeks later (**b**). Both myocardial contrast echocardiography (MCE) examinations showed homogeneous and equal enhancement intensity in the basal, middle and apical segments in the four-chamber view

In most instances, TCM is observed after emotional or physical stress; a recent paper also suggested an association between TCM and depression/mood disorders [3]. However, TCM can also be related to the stress of surgery, in which case, it is called perioperative TCM (pTCM) [4]. TCM seems to mostly affect postmenopausal female due to influences of female hormones on sympathetic neuromodulation and endothelial function [5]. In general, TCM patients had a comparable long-term mortality risk with Acute Coronary Syndrome, but a recent paper suggests the prognosis varies with triggers [6].

In most cases, coronary angiography, computed tomography angiography, cardiac magnetic resonance and radionuclide scans are used to exclude coronary heart disease. We present a case of postoperative TCM in a 51-year-old woman who underwent cardiac surgery. Intact microvascular myocardial perfusion was observed in myocardial contrast echocardiography (MCE) despite apical akinesis. This report supports the use of MCE as a substitute for invasive coronary angiography.

## Case presentation

A 51-year-old woman was admitted to our hospital complaining of chest tightness, palpitations and dyspnoea after activity. On admission, physical examination revealed a normal state of consciousness, an average heart rate of 76 beats per minute (bpm), and a blood pressure of 111/70 mmHg. Cardiac auscultation revealed variable first heart sound intensity and a diastolic murmur in the mitral stethoscope area. The other findings were unremarkable. The electrocardiogram showed atrial fibrillation. The echocardiogram performed by our hospital also showed rheumatic heart disease (severe mitral stenosis and regurgitation) with normal cardiac function and wall motion.

After the completion of the preoperative examination, the patient was sent to the operating room for mitral valve replacement. The operation was successful. After the aorta was reopened, ventricular fibrillation occurred. Sinus rhythm was not restored until 5 rounds of electrical defibrillation had been performed. Not long after returning to the intensive care unit (ICU), this patient developed heart failure with low blood pressure (70~85/45~50 mmHg) and tachycardia (125–135 bpm). Blood gas analysis showed progressive lactic acidosis, and blood lactate increased from 2.4 mmol/L to 15.3 mmol/L. Troponin T was slightly elevated compared to the preoperative level (1.960 ng/mL vs 0.019 ng/mL). N-terminal pro-brain natriuretic peptide levels increased markedly from 821.7 pg/mL to 21,025 pg/mL. Electrocardiogram (ECG) (Fig. 1) showed that the V5–6 ST-segment depression was 0.1 mV. The bedside chest film showed a small amount of fluid in the left chest. Urgent bedside echocardiography demonstrated akinesis in the middle and apical segments of the left ventricle with depressed LV function (EF 36%),while basal segments’ movement were generally normal.

To determine the blood flow in the myocardium, myocardial contrast echocardiography (MCE) was performed immediately (Figs. 2a, b, and 3a). For the MCE examination, the ultrasound system was switched to the contrast mode, with a mechanical index of 0.1–0.5. MCE was performed using intravenous administration of 2.0 mL of SonoVue (Bracco, Milan, Italy). To achieve a balance among ultrasound intensity, penetration, and the duration of myocardial opacification, the contrast-specific imaging mode needed to be adjusted. Similar enhancement intensity was observed in the basal, middle and apical segments. Quantitative analysis also showed approximately equivalent maximum intensity values in these regions.

The diagnosis was considered TCM instead of myocardial infarction. The treatment mainly involved maintaining effective circulation and reducing the postcardiac load. After the administration of epinephrine and norepinephrine, the patient’s blood pressure was still low. Considered the patient’s condition as heart failure, an intra-aortic balloon pump was inserted. Although myocardial enzyme levels were elevated, the doctors did not perform treatment for coronary heart disease, considering the reason of tissue injury after heart surgery. The therapeutic regimen for this patient included ventilator-assisted ventilation, postoperative anticoagulation therapy, anti-infection treatment and other conservative treatments.

A week later, the patient underwent coronary angiography, and the results showed no significant narrowing of the coronary artery. This patient was extubated 13 days after surgery and was later weaned from epinephrine. Myocardial contrast echocardiography performed 2 weeks later (Figs. 2c, d, and 3b) showed that apical movement was significantly improved, with a slight decrease in interventricular septal motion. In addition, the perfusion of the myocardium was normal, with an EF (Simpson) of 52%.

The clinical evolution was favourable, and the patient was discharged 3 weeks later. Before discharge, echocardiography showed that the artificial mitral valve function and the segmental wall movement were normal, with an EF of 72%. One year later, the patient remained asymptomatic and showed normalization of ventricular wall motion in the apical segment.

## Discussion

In this study, TCM was diagnosed in a middle-aged female patient who had slightly elevated myocardial enzyme levels, ECG abnormalities, normal epicardial coronary arteries, typical apical ballooning in echocardiography and normal myocardial perfusion in MCE according to the 2008 revised Mayo Clinic criteria [7]. The improvement of the left ventricular systolic dysfunction within 2 weeks was consistent with TCM.

We assessed the perfusion of the myocardium by using noninvasive MCE, which indicated no microcirculatory dysfunction. In contrast to ST-segment elevation myocardial infarction, TCM is characterized by a greater elevation in brain natriuretic peptide and by less myonecrosis [8]. Moreover, the abnormal wall motion of the patient’s left ventricle was not consistent with the blood flow distribution of an occluded epicardial coronary artery. The level of the biomarker troponin T was not proportional to the involved myocardial area [9]. In addition, MCE revealed no perfusion defects, which was consistent with the results of coronary angiography. Therefore, we believe that the integrity of the microvasculature can be revealed and coronary blood flow abnormalities can be excluded by performing MCE.

Some studies have used MCE to assess the perfusion of the myocardium in TCM patients. Studies have shown that compared with patients with acute myocardial infarction (AMI), patients with TCM have better preserved myocardial perfusion, as measured by MCE [10, 11]. Additionally, in some rat model experiments, apical perfusion has not been found to be impaired in the early phase of TCM [12]. Our case also showed good perfusion in MCE despite the regional wall motion abnormalities in the acute phase of TCM, which confirmed that coronary microcirculation was normal. In Abe’s study of 17 cases with TCM, a Doppler guidewire was used in 13 patients to assess coronary flow velocities [13]. In addition, MCE was used to assess the microcirculation of 1 patient. The examinations all showed normal coronary blood flow and were considered evidence of normal myocardial microcirculation in these patients. Hence, real-time MCE may be a useful and noninvasive diagnostic tool for differentiating TCM from AMI.

In most circumstances, emotional and physical stressors are thought to be the triggers for TCM. In recent years, perioperative factors have also attracted much attention as triggers of TCM [14–16]. In some cases, various perioperative factors, including intubation and extubation, radiofrequency ablation of atrial fibrillation in the region of the pulmonary veins, haemodynamic changes during the perioperative period, and excessive CO_2_ absorption, have been considered to contribute to the development of TCM by elevating catecholamine levels [1]. In this case, the patient had a history of atrial fibrillation. During the surgery, radiofrequency ablation was performed to disconnect the pulmonary vein. Blood gas analysis showed progressive lactic acidosis. All of these factors contributed to the occurrence of TCM in this patient.

## Conclusion

TCM is a stress-induced transient ventricular dysfunction for which the prognosis is usually good, but there are still some patients with severe symptoms that can be life threatening. In surgical departments (not limited to cardiovascular surgery departments), when patients have symptoms similar to myocardial infarction along with arrhythmia or other risk factors, the possibility of TCM might be considered. It is vital that we identify this condition in patients at the time of onset and provide mechanical circulatory support devices. MCE is a powerful, safe and noninvasive method for early detection of abnormal ventricular wall movement and local microcirculation abnormalities and can be performed as an alternative to invasive coronary angiography. Based on the advantages mentioned above, MCE might also be used for regular TCM follow-up examinations.

## Abbreviations

AMI: Acute myocardial infarction
bpm: Beat per minute
CAD: Coronary artery disease
ECG: Electrocardiogram
EF: Ejection fraction
ICU: Intensive care unit
LV: Left ventricular
MCE: Myocardial contrast echocardiography
pTCM: Perioperative takotsubo cardiomyopathy
TCM: Takotsubo cardiomyopathy
